# Supplementary material for: Combined quantification of intracellular (phospho-)proteins and transcriptomics from fixed single cells
Source: Sci Rep. 2019 Feb 6;9:1469. doi: 10.1038/s41598-018-37977-7 (PMC6365588; doi:10.1038/s41598-018-37977-7)
Supplement: Supplementary file 1 — Supplementary Information [file 41598_2018_37977_MOESM1_ESM.pdf]

Supplementary Information

Combined quantification of intracellular (phospho-)proteins and transcriptomics from fixed single cells.

Jan. P. Gerlach[1,2], Jessie A. G. van Buggenum[1], Sabine E.J. Tanis[1], Mark Hogeweg[1], Branco M. H. Heuts[1], Mauro J. Muraro[3], Lisa Elze[1], Francesca Rivello[4], Agata Rakszewska[4], Alexander van Oudenaarden[3], Wilhelm T. S. Huck[4], Hendrik G. Stunnenberg[2] and Klaas W. Mulder[1,*]

[1]Department of Molecular Developmental Biology, Radboud Institute for Molecular Life Sciences, Science Faculty, Radboud University, PO Box 9101, 6500 HB, Nijmegen, The Netherlands.

[2]Department of Molecular Biology, Radboud Institute for Molecular Life Sciences, Science Faculty, Radboud University, PO Box 9101, 6500 HB, Nijmegen, The Netherlands.

[3]Oncode Institute, Hubrecht Institute-KNAW (Royal Netherlands Academy of Arts and Sciences), Utrecht, the Netherlands.

[4]Radboud University, Institute for Molecules and Materials , Heyendaalseweg 135 , 6525 AJ Nijmegen , The Netherlands.

[*]Corresponding author (k.mulder@science.ru.nl)

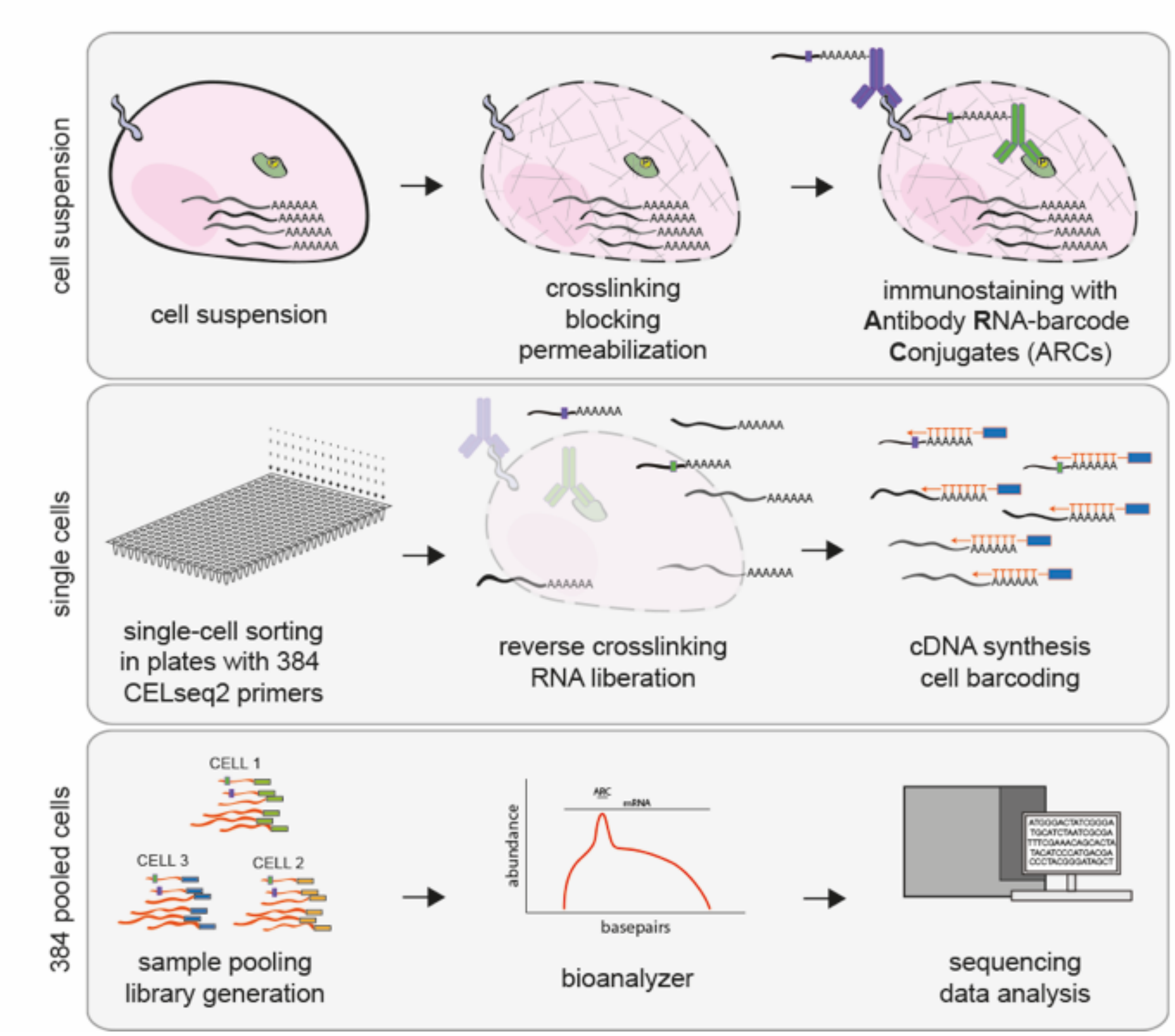

Figure S1. Detailed overview of the RAID procedure. Cells are collected and crosslinked in suspension using the reversible crosslinkers DSP and SPDP. Hereafter, cells are permeabilized using 0.1X Triton X100 and blocked using Protein Free Blocking buffer. Immunostaining with Antibody RNA-Barcode Conjugates (ARCs) is performed overnight. After extensive washing of the cells, they are sorted into 384-wells plates containing unique CELseq2 compatible primers, overlaid with mineral oil. To allow efficient lysis and reverse crosslinking, cells are first frozen at -80, followed by the addition of a DTT containing reverse crosslinking buffer. Next, reverse transcription is performed which incorporates a specific barcode in the cDNA from each cell and therefore allows sample pooling. Sequencing library preparation is performed according to an adapted CELseq2 protocol to allow efficient incorporation of ARC signals. The final sequencing library is composed of a broad mRNA signal and a specific ARC peak.

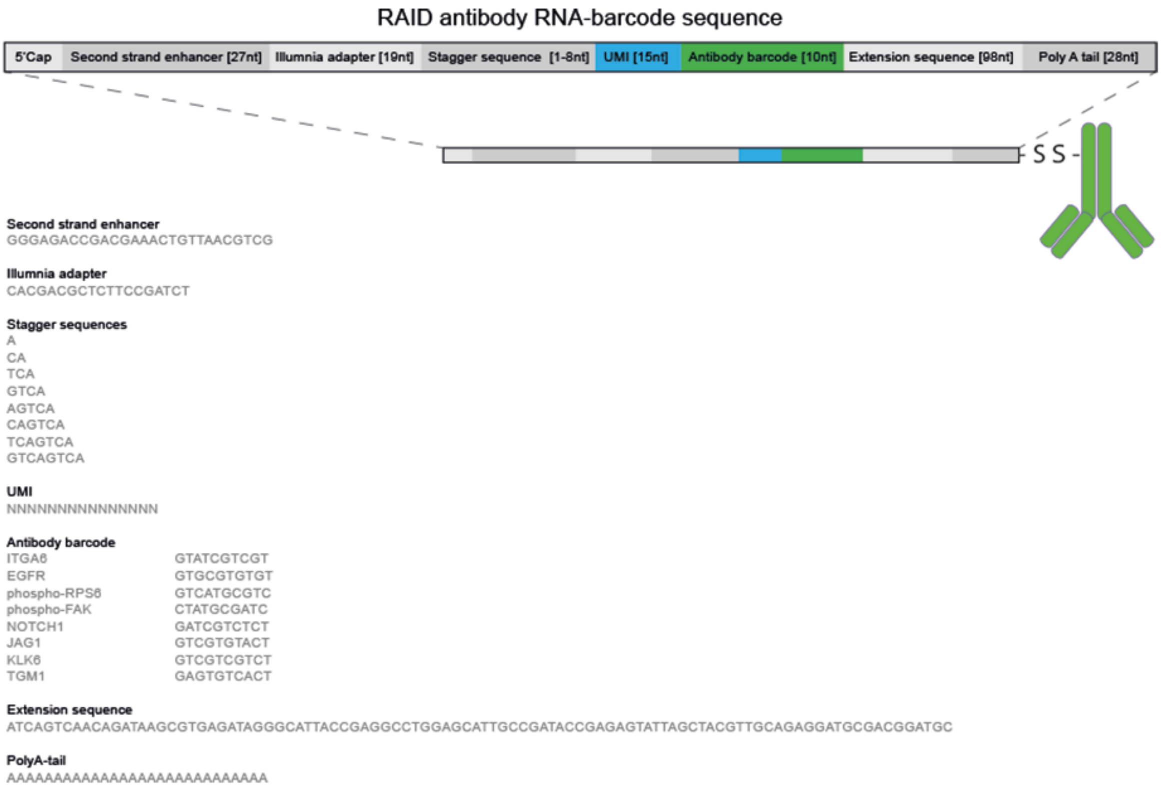

Figure S2. Sequence overview of the RAID antibody RNA-barcodes. The RNA barcode contains a 5’ Cap which is incorporated during the *in vitro* transcription based barcode production with the goal to enhance barcode stability and resemblance to mRNAs. The second strand enhancer is a sequence that is used to enhance priming of the second strand synthesis for RNA-barcodes. The Illumina adapter is part of the complete adapter sequence required for sequencing. The stagger sequence is a sequence of barcode dependent length that aims to prevent sequence failure due to overrepresentation of a common barcode sequence. The UMI within the RNA-barcode is use for the quantification of the ARCs. The antibody barcode sequence is the antibody specific sequence that allows antibody identification and ARC multiplexing. The RNA-barcode includes an extension sequence that enhances the efficiency of the CELseq2 based library prep for the ARCs. Finally, the RNA-barcodes contain a 28nt polA tail to mimic cellular mRNAs and allow CELseq2 based library preparation.

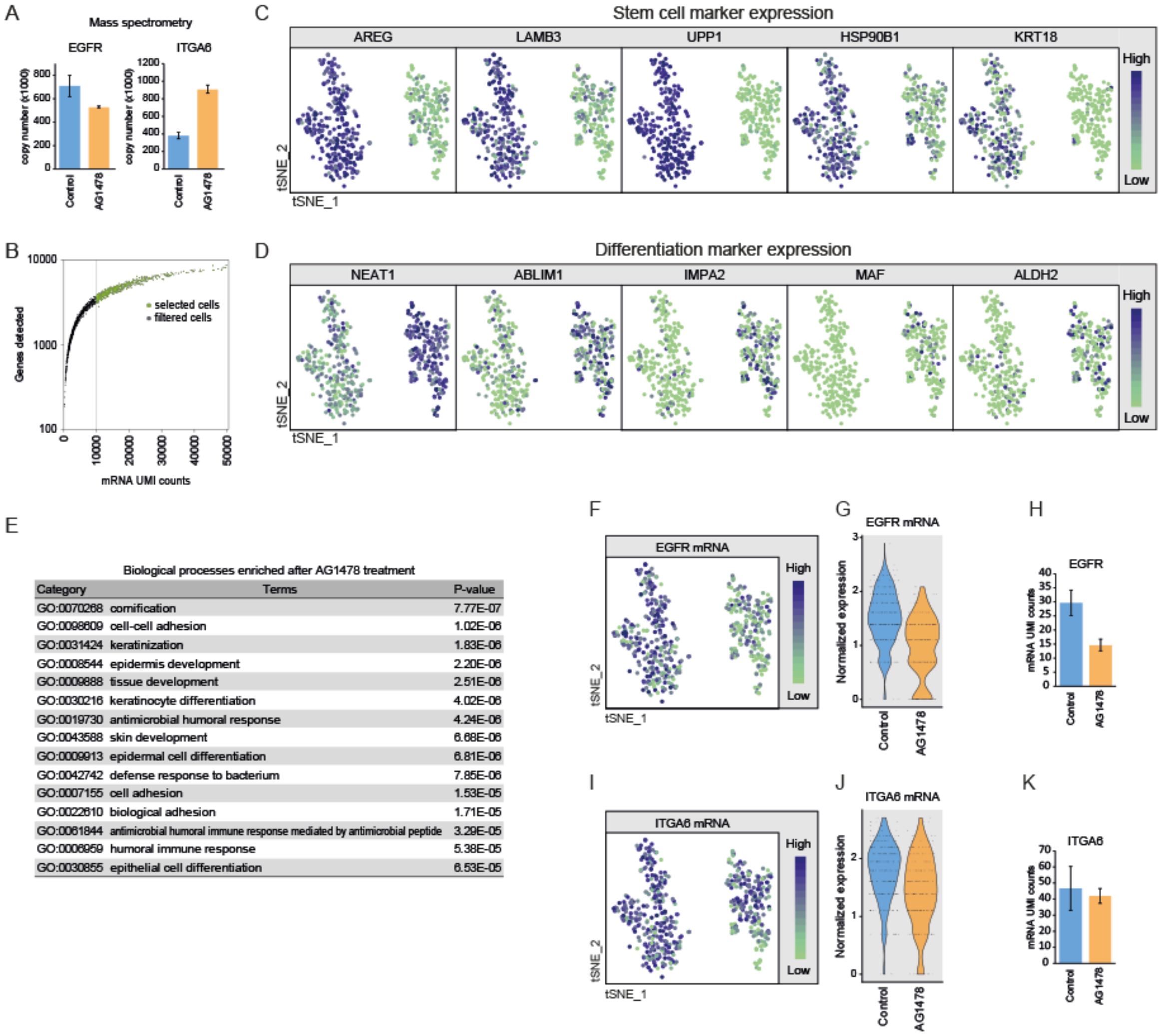

Biological processes enriched after AG1478 treatment

Category	Terms	P-value
GO:0070268	cornification	7.77E-07
GO:0098609	cell-cell adhesion	1.02E-06
GO:0031424	keratinization	1.83E-06
GO:0008544	epidermis development	2.20E-06
GO:0009888	tissue development	2.51E-06
GO:0030216	keratinocyte differentiation	4.02E-06
GO:0019730	antimicrobial humoral response	4.24E-06
GO:0043588	skin development	6.68E-06
GO:0009913	epidermal cell differentiation	6.81E-06
GO:0042742	defense response to bacterium	7.85E-06
GO:0007155	cell adhesion	1.53E-05
GO:0022610	biological adhesion	1.71E-05
GO:0061844	antimicrobial humoral immune response mediated by antimicrobial peptide	3.29E-05
GO:0006959	humoral immune response	5.38E-05
GO:0030855	epithelial cell differentiation	6.53E-05

Figure S3. Additional data to Figure 2, Combined single-cell transcriptomics and Antibody RNA-barcode Conjugate (ARC) detection from unfixed keratinocytes. (A) Boxplots showing the estimated copy numbers of EGFR and ITGA6 proteins in mass-spectrometry based proteomics of untreated and AG1478 treated Keratinocytes. The experiment was performed in triplicate. (B) Scatterplot showing the number of genes detected per cell, related to the total UMI counts of the transcriptome. Cells that passed the count thresholds for the mRNA (10000) and ARCs (2750) are indicated in green. (C) Featureplots showing the normalized mRNA expression of a selection of stem cell markers projected on the tSNE coordinates from Figure 2C. (D) Featureplots showing the normalized mRNA expression of a selection of differentiation markers projected on the cells of the tSNE coordinates from Figure 2C. (E) GO analysis shows biological processes significantly upregulated after AG1478 induced differentiation. **(**F, G) Normalized EGFR mRNA expression projected on the tSNE embedding from Figure 2C (F) and represented in violin plot (G). (H) Boxplots showing the EGFR mRNA

expression in bulk analysis of untreated and AG1478 treated keratinocytes. The experiment was performed in triplicate. (I, J) Normalized ITGA6 mRNA expression projected on the tSNE embeddings from Figure 2C (I) and represented in violin plot (J). (K) Boxplots showing the ITGA6 mRNA expression in bulk analysis of untreated and AG1478 treated keratinocytes. The experiment was performed in triplicate.

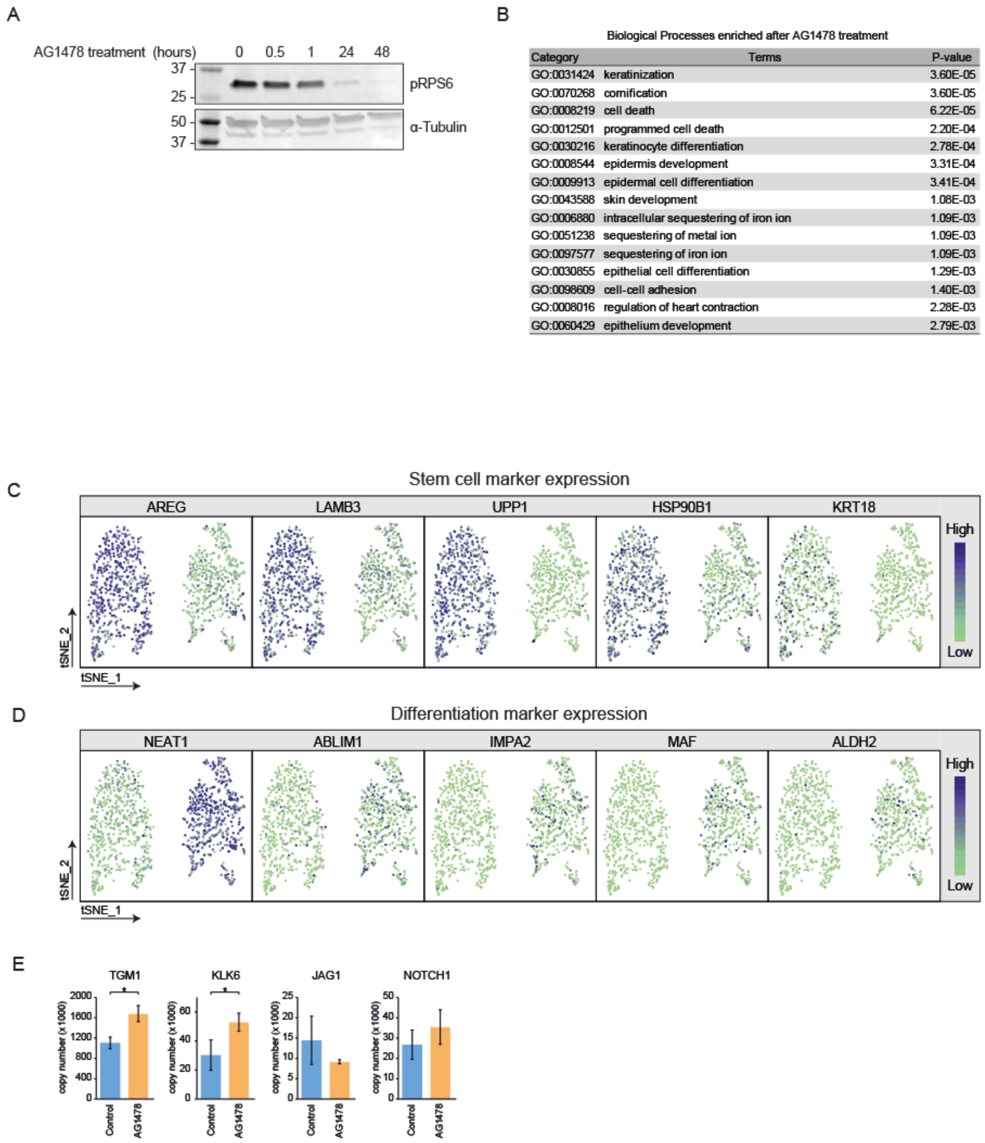

Biological Processes enriched after AG1478 treatment

Category	Terms	P-value
GO:0031424	keratinization	3.60E-05
GO:0070268	cornification	3.60E-05
GO:0008219	cell death	6.22E-05
GO:0012501	programmed cell death	2.20E-04
GO:0030216	keratinocyte differentiation	2.78E-04
GO:0008544	epidermis development	3.31E-04
GO:0009913	epidermal cell differentiation	3.41E-04
GO:0043588	skin development	1.08E-03
GO:0006880	intracellular sequestering of iron ion	1.09E-03
GO:0051238	sequestering of metal ion	1.09E-03
GO:0097577	sequestering of iron ion	1.09E-03
GO:0030855	epithelial cell differentiation	1.29E-03
GO:0098609	cell-cell adhesion	1.40E-03
GO:0008016	regulation of heart contraction	2.28E-03
GO:0060429	epithelium development	2.79E-03

Figure S4. Additional data to Figure 4. RAID analysis shows association of FAK phosphorylation with the expression of stem cell marks. (A) AG1478 treatment triggers loss of phosphorylated RPS6 as shown by western blotting and immunodetection. (B) GO analysis shows biological processes significantly upregulated in RAID cells after AG1478 induced differentiation. (C) Featureplots showing the normalized mRNA expression of a selection of stem cell markers projected on the cells of the tSNE from Figure 4A. (D) Featureplots showing the normalized mRNA expression of a selection of differentiation markers projected on the cells of the tSNE from Figure 4A. (E) Boxplots showing the estimated cellular copy numbers of TGM1, KLK6, JAG1 and NOTCH1 proteins in mass-spectrometry based proteomics of untreated and AG1478 treated keratinocytes. The experiment was performed in triplicate. Significant differences between untreated and AG1478 treated cells are indicated by asterisk (2-tailed t-test $p<0.05$).

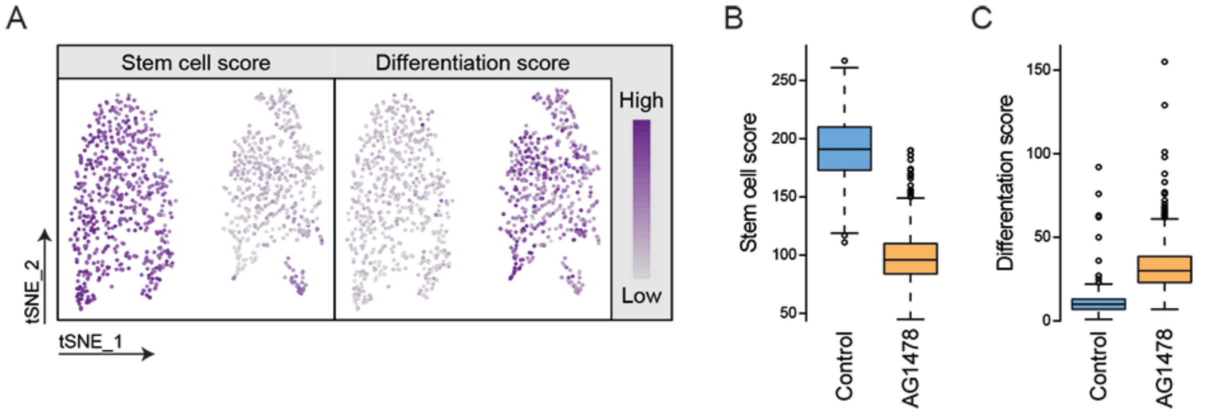

Figure S5. Stem cell and differentiation scores summarize the differentiation status of keratinocytes. (A) Stem cell and differentiation scores projected on the tSNE plot from Figure 4A. (B) Boxplots showing the stem cell scores of untreated and AG1478 treated RAID cells (C) Boxplots showing the differentiation scores of untreated and AG1478 treated RAID cells.

Detailed RAID Library Preparation Protocol for Keratinocytes

Jan Patrick Gerlach, version 2018-05-15

Materials:

Ordering of specific materials:

- 384-well PCR plates (Biorad, HSP3801)
- Aluminium foil seals for 384-well plates (VWR, 391-1281)
- Mineral Oil (Sigma-Aldrich, M5310-1L)
- Exonuclease I (E. coli) (NEB, M0293L)
- Maxima H minus Reverse Transcriptase (Thermo scientific, EP0753)
- RNAsin Plus (Promega, N2615)
- 1.5 ml LoBind tubes (Eppendorf, 022431021)
- 5X Second Strand Buffer (Thermo Scientific, 10812-014)
- DNA ligase (E. coli) (NEB, M0205L)
- DNA Polymerase I (E. coli) (NEB, M0209L)
- rSAP (NEB, M0371S)
- RNase H (Ambion, AM2293)
- MEGAscript T7 Transcription kit (Ambion, AM1334)
- Ampure XP Beads (Beckman Coulter, A63882)
- Herculase II Fusion Enzyme (Agilent, 600679)

Prepare in advance:

- 10 mM dNTP mix
- 1 M Tris-HCl pH 8
- 1 M DTT, keep frozen
- 10 % Triton X-100
- 5X RT-Buffer *(home-made without DTT)* [ ]
- Beads Buffer [20 % PEG8000, 2.5 M NaCl]

General procedures:

- Low volumes are dispensed in 384-well plates with the Nanodrop2 (BioNex)
- LoBind tubes are used until the libraries are amplified by *in vitro* transcription (IVT). After IVT conventional tubes are used.
- 384-well plates were centrifuged for 2 minutes at 1000 xg.

Primer sequences:

- CEL-Seq2 primers:
  5' GCCGGTAATACGACTCACTATAGGGGTTCAGACGTGTGCTCTTCCGATCTNNNNNNNNCGTCTAATTTTTTTTTTTTTTTTTTTTTTTTTV
- ❖ Cell barcode sequence (highlighted) is variable. For a full list of barcodes view the supplemental file F5.

- Barcode compensation primer:
  5' GGGAGACCGACGAAACTGTTAACG

- FWD random octamer:
  5' CACGACGCTCTTCCGATCTNNNNNNNN

- FWD pre-amplification primer:
  5' CACGACGCTCTTCCGATCT

- REV pre-amplification primer:
  5' GTTCAGACGTGTGCTCTTCCGATC

- FWD Library primer: (compatible with Illumina sequencing)
  5' AATGATACGGCGACCACCGAGATCTACACTCTTTCCCTACACGACGCTCTTCCGATCT

- REV Library indexing primer: (compatible with Illumina sequencing)
  5' CAAGCAGAAGACGGCATACGAGATATCAGTGTGACTGGAGTTCAGACGTGTGCTCTTCCGATC
- ❖ Illumina Index sequence (highlighted) is variable.

Preparation of 384-well plates

- Pipette 5 µl of mineral oil in 384-well plates
- Dispense 100 nl of (7.5 ng/µl) CEL-Seq2 primers in the 384-well plates
- Centrifuge and store at -20 °C until ready for sorting

Single cell sorting

- Single cells were sorted into the 384 wells plates
- Spin down plated for 5 minutes at 1000 xg
- Freeze at -80 °C

Reverse cross-linking

- Thaw the plates
- Add 50 nl of reverse cross-linking additive (prepare fresh) and spin down

Reverse cross-linking additive

-	10 mM dNTP	30 nl	13.5 µl
-	1 M Tris pH 8	7.5 nl	3.375 µl
-	1 M DTT	4.5 nl	2.025 µl
-	10 % TX-100	0.5 nl	0.225 µl
-	RNAsin Plus	7.5 nl	3.375 µl
	Stock materials	*per well (50 nl)*	*per plate (x450) = 22.5 µl*

❖ Prepare reverse cross-linking mixture for as many plates as required + 80 µl for dead volume.

- Incubate in 384-well PCR machine:
  - 45 minutes at 25 °C
  - 5 minutes at 65 °C
  - continuous 4 °C

Reverse transcription (RT)

- Prepare the RT-Enzyme Mixture as indicated below

RT-Enzyme Mix

- 5X RT-Buffer *(home-made, without DTT)*	0.05 µl)	22.5 µl
- 10 mM dNTP	0.01 µl)	4.5 µl
- H_2O	0.0125 µl)5.625 µl	
- Maxima H minus RT enzyme	0.02 µl)	9 µl
- RNAsin Plus	0.0075 µl)3.375 µl	
Stock materials	*per well (100 nl)*	*per plate (x450) = 45 µl*

- Dispense 100 nl in each well and spin down
- Perform the RT reaction:
  - 10 minutes at 25 °C
  - 60 minutes at 50 °C
  - 10 minutes at 85 °C
  - continuous 4 °C

- Dispense 50 nl of 0.3 pmol/µl Barcode compensation primer solution in the wells and centrifuge
- Incubate in 384-well PCR machine
  - 5 minutes at 85 °C
  - decrease temperature 5 degrees and incubate 30 seconds
  - repeat previous step until samples are at 10 °C

❖ Stopping point – samples can be stored @ -20 °C

First Strand cDNA pooling and Exonuclease (EXOI) treatment

- Collect and pool the samples per plate
- Pipette off and discard as much as possible from the upper (mineral oil) phase without disturbing the lower phase
- Collect the (lower) water phase by piercing through the remaining layer of mineral oil and transfer to new tube

❖ It is crucial not to have any mineral oil in the final tube since this can disturb the beads purification.

- Measure the collected aqueous phase and add up to 91 µl with water, to level all samples
- Add 5 µl EXOI and incubate for 20 minutes at 37 °C

First Strand cDNA cleanup

- Prewarm Ampure XP Beads to room temperature
- Add 35 µl Ampure XP Beads + 157 µl Beads Buffer to the samples and transfer to 1.5 ml LoBind Tubes
- Incubate at room temperature for 15 min
- Place on magnetic stand for at least 5 min, until liquid appears clear
- Remove and discard the supernatant
- Add 200 µl freshly prepared 80 % Ethanol
- Incubate 30 seconds
- Add 200 µl freshly prepared 80 % Ethanol

- Incubate 30 seconds
- Air dry beads until completely dry
- Resuspend with 15 µl water. Pipette entire volume up and down to mix thoroughly
- Incubate at room temperature for 5 min
- collect 15 µl in a clean PCR tube
- ❖ Stopping point – Samples can be stored at -20 °C

Second Strand RT reaction

- Move previous step to ice so it cools below 16 °C
- Add 5.1 µl of the Second Strand Mix to each reaction tube:

Second Strand Mix		
- Second Strand Buffer	3.875 µl	
- dNTP (10 mM)		0.3875 µl
- DNA ligase		0.1395 µl
- DNA Pol I		0.5425 µl
- RNaseH		0.1395 µl
		per sample

- Incubate at 16 °C for 120 minutes

Second Strand cDNA cleanup:

- Prewarm Ampure XP Beads to room temperature
- Add 33 µl Ampure XP Beads to the samples and transfer to 1.5 ml LoBind Tubes
- Incubate at room temperature for 15 min
- Place on magnetic stand for at least 5 min, until liquid appears clear
- Remove and discard the supernatant
- Add 190 µl freshly prepared 80 % Ethanol
- Incubate 30 seconds
- Add 190 µl freshly prepared 80 % Ethanol
- Incubate 30 seconds
- Air dry beads until completely dry
- Resuspend with 6.5 µl water. Pipette entire volume up and down to mix thoroughly
- Incubate at room temperature for 5 min
- collect 6 µl in a clean PCR tube
- ❖ Stopping point – Samples can be stored at -20 °C

IVT using Ambion T7 MEGA-Script kit:

- Prepare the IVT Reaction mix and add 11 µl per sample

IVT Reaction Mix	
- ATP	1.7 µl
- TTP	1.7 µl
- CTP	1.7 µl
- GTP	1.7 µl
- 10x T7 buffer	1.7 µl
- T7 enzyme	1.7 µl
- RNAsin plus (not part of kit)	0.8 µl
	per sample

- Incubate in a thermal cycler at 37 °C for 14 hours, with lid at 50 °C. Set cycler to go to 4 °C at end of incubation.

EXOI / rSAP treatment:

- Add 0.5 µl EXOI to the samples
- Add 0.5 µl rSAP to the samples
- Incubate at 25 minutes at 37 °C

Amplified RNA cleanup:

- Prewarm Ampure XP Beads and Beads Buffer to room temperature
- Vortex Ampure XP Beads until well dispersed, add 32.4 µl to the samples and transfer to room temperature
- Incubate at room temperature for 15 min
- Place on magnetic stand for at least 5 min, until liquid appears clear
- Remove and discard the supernatant
- Add 200 µl freshly prepared 80 % Ethanol
- Incubate at least 30 seconds, then remove and discard supernatant without disturbing beads
- Repeat this wash step two more times
- Air dry beads until completely dry
- Resuspend with 12 µl water
- Incubate at room temperature for 10 min
- Place on magnetic stand for 5 min, until liquid appears clear
- Collect 5 µl and store rest at -80 °C

Library Prep Reverse Transcription reaction:

- To 5 µl amplified RNA add:
  - 1 µl primer mixture
    (= 0.2 µl of 5 pmol/µl Barcode Compensation primer + 0.8 µl of 5 pmol/µl FWD random_octamer)
  - 1 µl 10 mM dNTPs
- ❖ The ratio between Barcode Compensation primer and FWD random_octamer can be adapted if required. Furthermore, separate libraries enriched for Antibody-Barcodes or mRNA can be obtained by performing two RT reaction with either Barcode Compensation primers and FWD random_octamers, respectively.
- Incubate 3 min at 65 °C, quick chill on ice
- Prepare RT-Enzyme Mix

**RT-Enzyme Mix**	
- 5x First Strand buffer	2 µl
- RNasin	0.5 µl
- Maxima RT enzyme	0.5 µl
	per sample

- Add 3 µl of the RT-Enzyme Mix to each reaction sample:
- Run Reverse Transcription protocol:
  - 10 min at 25 °C.
  - 60 min at 50 °C
  - 10 min at 85 °C
  - continuous at 4 °C
- ❖ Stopping point – Samples can be stored at -20 °C

Library Pre-amplification

- To the 10 µl RT mixture of each sample, add 15 µl of pre-amplification PCR mix:

**Pre-amplification PCR Mix**	
- Ultra-Pure Water	8 µl
- 5X Herculase II reaction buffer	5 µl
- dNTP (from Herculase II kit)	0.5 µl
- FWD preamp. primer (5 pmol/µl)	0.5 µl
- REV preamp. primer (5 pmol/µl)	0.5 µl
- Herculase II polymerase	0.5 µl
	per sample

- Amplify using the following PCR conditions:
  - 30 seconds at 95 °C
  - 12 cycles of:
    - 30 seconds at 95 °C
    - 30 seconds at 60 °C
    - 60 seconds at 72 °C
  - 5 minutes at 72 °C
  - Continuous at 4 °C
- ❖ Stopping point – Samples can be stored at -20 °C

EXOI treatment

- Add 0.5 µl EXOI and incubate 20 minutes at 37 °C

PCR cleanup:

- Prewarm RNAClean XP beads and Beads Buffer to room temperature
- Vortex Ampure XP beads until well dispersed, add 26 µl to the samples and transfer to room temperature
- Incubate at room temperature for 15 min
- Place on magnetic stand for at least 5 min, until liquid appears clear
- Remove and discard the supernatant
- Add 200 µl freshly prepared 80 % Ethanol
- Incubate at least 30 seconds, then remove and discard supernatant without disturbing beads
- Repeat this wash step one more time
- Air dry beads until completely dry
- Resuspend with 15 µl water
- Incubate at room temperature for 10 min
- Place on magnetic stand for 5 min, until liquid appears clear
- Collect 15µl in clean PCR tube

Library Prep. PCR

- To 15 µl preamplified sample, add 9 µl Library Prep PCR mix and 1 µl of a unique REV Library indexing primer

<u>Library Prep PCR Mix</u>

- Ultra-Pure Water	2.5 µl
- 5X Herculase II buffer	5 µl
- dNTP (from Herculase II kit)	0.5 µl
- FWD Library primer (5 pmol/µl)	0.5 µl
- Herculase II polymerase	0.5 µl
	per sample

- To each reaction add 1 µl of a unique REV Library indexing primer (2.5 pmol/µl) to index all the samples on the Flow Cell

- Amplify using the following PCR conditions:
  - 30 seconds at 95 °C
  - 6 cycles of:
    - 30 seconds at 95 °C
    - 30 seconds at 60 °C
    - 45 seconds at 72 °C
  - 5 minutes at 72 °C
  - Continuous at 4 °C

❖ Stopping point – Samples can be stored at -20 °C

EXOI treatment

- Add 0.5 µl EXOI and incubate 20 minutes at 37 °C

Purification and size selection

- Add 20.5 µl Ampure XP Beads to the samples
- Incubate at room temperature for 15 min
- Place on magnetic stand for at least 5 min, until liquid appears clear
- remove supernatant
- Add 190 µl freshly prepared 80 % Ethanol to the beads
- Incubate 30 seconds
- Add 190 µl freshly prepared 80 % Ethanol
- Incubate 30 seconds
- Air dry beads until completely dry
- Resuspend in 15 µl water. Pipette entire volume up and down to mix thoroughly
- Incubate at room temperature for 5 min
- Collect samples in a clean LoBind Eppendorf tube

❖ Library prep is now done. The samples should be analyzed by Qbit and Bioanalyzer before sequencing.

Detailed RAID Fixation and Staining Protocol for Keratinocytes

Jan Patrick Gerlach, version 2018-06-11

Materials:

Ordering of specific materials:

- DSP (*dithiobis(succinimidyl propionate)*) (Thermo scientific, 22585)
- SPDP *(succinimidyl 3-(2-pyridyldithio)propionate)* (Thermo scientific, 21857)
- PFBB (*Protein-Free Blocking Buffer in PBS)* (Thermo scientific, 37572)
- tRNA (Roche, 10109495001)
- RNAsin Plus (Promega, N2615)
- 1.5ml LoBind tubes (Eppendorf, 022431021)

Prepare in advance:

- Na-PS Buffer: [200 mM Sodium-Phosphate buffer pH 8.4, 150 mM NaCl] To prepare 1 M stock solution of Sodium-Phosphate buffer, mix 1 M NaH_2PO_4 and 1 M Na_2HPO_4 solutions to set pH to 8,4
- 50mM DSP stock solution: [Dissolve DSP in anhydrous DMSO, store at -20 °C]
- 50mM SPDP stock solution: [Dissolve SPDP in anhydrous DMSO, store at -20 °C]
- Quench Buffer: [100 mM Tris-HCl pH 7.5, 150 mM NaCl]
- Wash Buffer [1X PFBB diluted 1:10 with PBS]

General procedures:

- Cells were centrifuged 5 minutes at 400 xg, unless indicated otherwise

Cell collection:

- ➢ Collect all cells, count and pellet (5 minutes at 1000 xg in a 50 ml Tube)
- ➢ Resuspend cells in PBS at 1 M/ml
- ➢ Collect 5M cells per condition in 15ml tubes
- ➢ Centrifuge and aspirate supernatant

DSP/SPDP Fixation:

- ❖ Skip this section for cell surface staining of unfixed cells
- ➢ Resuspend cells in 2.5 ml Na-PS Buffer
  - Prepare 2.5 ml 2X Fixative solution per sample, add immediately to the cell suspension and resuspendMix 250 µl 50 mM DSP and 250 µl 50 mM SPDP in an empty tube, add 2 ml Na-PS Buffer, mix briefly and immediately add to the cells
  - ❖ DSP and SPDP are hydrolyzed with very short half-life when in contact with aqueous solutions. Add solution to cells without pause.
- ➢ Incubate for 45 minutes at room temperature while gently rolling the tubes (longer incubation is not detrimental)
  - ❖ This is the best step to prepare the BP-Buffer (view RAID immunostain section)
- ➢ Centrifuge, aspirate supernatant and resuspend in 5 ml Quench Buffer
- ➢ Incubate for 10 minutes at room temperature
- ➢ Gently mix cells by pipetting up and down and filter out cell-clumps with a 70 µm filter
- ➢ Spin down only when ready for immunostain

RAID Immunostain:

- ➢ Prepare 1 ml BP-Buffer per sample:

  BP-Buffer:

  Dilute PFBB 1:1 with PBS, supplement with 100 µg/ml of tRNA (pre-boiled), 0.5 U/µl RNAsin Plus, 0.1 % Triton X100
  - ❖ Exclude Triton X100 from the buffer for cell surface staining of unfixed cells
- ➢ Resuspend cells in 1 ml BP-Buffer and transfer to 1.5 ml LoBind tubes
- ➢ Incubate for 30 minutes gently rolling at room temperature
- ➢ Prepare 1 ml RAID Staining Buffer per sample:

  RAID Staining Buffer:

  Dilute PFBB 1:1 with PBS, supplement with 2 U/µl RNAsin Plus, 0.1 % Triton X100, 250 ng/µl (each) Antibody-Barcode conjugate
  - ❖ *Antibody concentrations may be adapted as required*
  - ❖ Exclude Triton X100 from the buffer for cell surface staining of unfixed cells
- ➢ Centrifuge, aspirate supernatant and resuspend in 1 ml RAID Staining Buffer
- ➢ Incubate for overnight (~20 hours) gently rolling at 4 °C
  - ❖ Incubate for 2 hours for cell surface staining of unfixed cells
- ➢ Centrifuge, aspirate supernatant and resuspend in 10 ml Wash Buffer
- ➢ Repeat this wash step 6 times, last 3 washes were incubated for 10 minutes

- Centrifuge, aspirate supernatant and resuspend in PBS at a concentration of approximately 1 M/ml
- Transfer to FACS tube and bring to the FACS facility for sorting
